# Self‐Induced A‐B‐A Structure Enables Efficient Wide‐Bandgap Perovskite Solar Cells and Tandems

**DOI:** 10.1002/advs.202413749

**Published:** 2025-03-05

**Authors:** Xixi Yu, Yong Zhu, Kunpeng Li, Xiong Chang, Mengni Zhou, Zhewen Xie, Xing Zhu, Hua Wang, Wenhui Ma, Shaoyuan Li, Tao Zhu

**Affiliations:** ^1^ Faculty of Metallurgical and Energy Engineering Kunming University of Science and Technology Kunming 650093 P. R. China; ^2^ State Key Laboratory of Complex Nonferrous Metal Resources Clean Utilization Kunming University of Science and Technology Kunming 650093 P. R. China; ^3^ School of Engineering Yunnan University Kunming 650500 P. R. China; ^4^ Yunnan Key Laboratory of Clean Energy and Energy Storage Technology Kunming 650093 P. R. China

**Keywords:** A‐B‐A structure, halide segregation, self‐induced, vertical crystal orientation, wide‐bandgap perovskite

## Abstract

Wide‐bandgap (WBG) perovskite solar cells (PSCs), due to their tunable bandgap, can be integrated into tandem cell configurations with narrow‐bandgap solar cells to overcome the shockley‐queisser (SQ) limitation. However, the main obstacles limiting their performance are poor crystallinity and light‐induced halide segregation. To achieve high performance in WBG PSCs, this study reports a dual‐molecule cooperative strategy involving the introduction of 1‐benzyl‐3‐methylimidazolium bromide (BzMIM Br) as an additive and the introduction of 6‐fluoropyrimidine‐2,4‐ diamine (DMFP) as a passivation layer. DMFP self‐induced penetration to the bottom of the perovskite, forming an A‐B‐A structure with BzMIM Br, through utilizing multisite integration with uncoordinated Pb^2+^, constructing internal molecular bridges. Research findings indicate that the A‐B‐A structure with uniform potential distribution can interact with the perovskite in a step‐like manner, suppressing halide segregation, and replenishing the vacancy defects. Results demonstrate power conversion efficiencies (PCEs) of 22.77% and 18.54% for inverted PSCs with effective areas of 0.043 and 1.0 cm^2^, respectively. Unencapsulated devices retain 95% of initial efficiency after 1500 h of continuous illumination under one‐sun equivalent conditions in a nitrogen atmosphere. Additionally, the PCE of the prepared semi‐transparent WBG devices reached 19.60%, while the PCE of the 4‐terminal all‐perovskite tandem device reached 26.18%.

## Graphical abstract

DMFP penetrates to the bottom of the perovskite, forming an A‐B‐A sandwich structure with BzMIM Br, leading to strong interactions with the perovskite. In the vertical direction, utilizing multisite coordination with Pb ions forms internal molecular bridges, which not only improves crystallographic orientation but also suppresses halide phase segregation in the vertical direction. Furthermore, the A‐B‐A structure, through vacancy defect filling, achieves uniform component modulation, leading to a significant enhancement in performance.

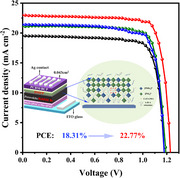

## 1 Introduction

Metal halide perovskites have attracted significant attention due to their high light absorption coefficient, tunable bandgap width, good low‐light performance, long carrier mobility, and simple preparation process.^[^ ^1^ ^]^ As one of the new generation photovoltaic technologies, single‐junction perovskite solar cells (PSCs) have achieved certified power conversion efficiencies (PCE) as high as 26.7%, approaching the highest certified PCE of commercial silicon solar cells (27.1%).^[^ ^2^ ^]^ However, the potential for further improvement in PCE is limited due to the limitations of the Shockley–Queisser (SQ) limit.^[^ ^3^ ^]^ Tandem solar cells (TSC) can effectively utilize photon energy, offering significant potential for overcoming the SQ limit of PSCs.^[^ ^4^ ^]^ To excite the potential of TSC, it is necessary to identify suitable wide‐bandgap (WBG) perovskite solar cells as the top cell of the TSC (Eg > 1.65 eV, where Eg represents the bandgap of the perovskite film),^[^ ^5^ ^]^ with narrow‐bandgap (NBG) cells serving as the bottom cell, thereby maximizing the utilization of photon energy.^[^ ^6^ ^]^ Narrow‐bandgap materials include mixed lead‐based and tin‐based perovskites,^[^ ^7^ ^]^ crystalline silicon,^[^ ^8^ ^]^ among others. Therefore, the development of efficient WBG PSCs is crucial for achieving high‐efficiency TSCs.

It is widely known that numerous strategies have been implemented to enhance the performance of wide‐bandgap perovskites, such as additive engineering,^[^ ^9^ ^]^ post‐treatment strategies,^[^ ^10^ ^]^ etc., addressing issues like halide segregation, severe open‐circuit voltage (V_oc_) losses, and crystallinity. In tackling the halide segregation issue, researchers have used large‐size cations (such as potassium (K^+^), rubidium (Rb^+^)) or partially replaced A‐site cations with organic–inorganic hybrids (such as cesium (Cs^+^)) to enlarge the bandgap and lower the Br/I ratio in the perovskite composition.^[^ ^11^ ^]^ For instance, Edward Sargent et al. doped Rb into the inorganic perovskite lattice, achieving a bandgap of ≈2.0 eV and a relatively stable perovskite phase while suppressing light‐induced halide segregation.^[^ ^12^ ^]^ Additionally, Chen et al. introduced 4‐(2‐aminoethyl)‐benzene sulfonyl fluoride salt with multifunctional groups (sulfonyl, ammonium, and fluoride) into mixed‐halide precursors, demonstrating that a downward uniform crystallization strategy can inhibit initial vertical halide segregation during perovskite crystallization and reduce V_oc_ losses.^[^ ^13^ ^]^ Severe V_oc_ losses primarily result from rapid perovskite crystallization leading to poor film quality.^[^ ^14^ ^]^ Zhang et al. employed a tailored solvent engineering strategy, replacing dimethyl sulfoxide with 1,3‐dimethyl‐3,4,5,6‐tetrahydro‐2(1H)‐pyrimidinone, effectively delaying and modulating the crystallinity and growth process of perovskite films, achieving high‐quality perovskite films and efficient perovskite solar cells with a wide solvent processing window and only 0.394V V_oc_ loss.^[^ ^15^ ^]^ While the introduction of large‐size A‐site cations, multifunctional groups, and other strategies can mitigate issues like light‐induced halide segregation, they inevitably introduce lead iodide vacancies and halide vacancy defects, increasing the defect density at grain boundaries. This affects carrier transport by imposing grain boundary resistance, significantly reducing extraction and transport rates.^[^ ^16^ ^]^ Therefore, addressing issues such as light‐induced halide segregation and poor crystallinity is crucial, along with the need to minimize the formation of defect states at grain boundaries to achieve high efficiency, high crystallinity, and higher‐quality PSCs.^[^ ^17^ ^]^ However, the literature reports only single‐molecule strategies for controlling perovskites, which do not allow simultaneous regulation of the upper interface, perovskite layer, and lower interface. Based on this, how to achieve stepwise regulation of perovskites remains to be studied.

In view of this, we have proposed an effective bimolecular cooperative regulation method by introducing 1‐benzyl‐3‐methylimidazolium bromide (BzMIM Br) as an additive in the WBG perovskite precursor and 6‐fluoropyrimidine‐2,4‐diamine (DMFP) as a passivation layer on the perovskite surface. DMFP self‐induces penetration to the bottom of the perovskite, combining with BzMIM Br to form an A‐B‐A structure with uniform potential distribution. This structure constructs internal molecular bridges by passivating uncoordinated Pb^2+^ through multisite, interacting in a step‐like manner with the perovskite, passivating positive and negative charge defects, controlling crystal orientation in the vertical direction, and effectively suppressing halide segregation, thus achieving large‐sized, high‐quality crystalline perovskite films. The research results demonstrate that inverted WBG PSCs based on 1.68 eV achieved a reproducible PCE of 22.77%, with a short‐circuit current density (J_sc_) reaching a record‐breaking 22.96 mA cm^−2^, placing them among the highest values for 1.68 eV WBG PSCs. Unencapsulated devices retained over 95% of initial efficiency after 1500 h of continuous illumination under one‐sun equivalent conditions in a nitrogen atmosphere. Furthermore, when applied to semi‐transparent devices and 4T tandem devices, the PCE reached 19.60% and 26.18%, respectively. Good performance is also achieved in 1 cm^2^ large‐area devices, with a PCE of 18.54%.

## 2 Results and Discussion

As shown in Figure S1a (Supporting Information), the molecular formulas of two molecules are presented: i) 6‐fluoropyrimidine‐2,4‐diamine (DMFP) and ii) 1‐benzyl‐3‐methylimidazolium bromide (BzMIM Br). The selection of these two substances as reactive materials for modifying perovskite films is primarily considered from two aspects. Firstly, DMFP contains a pyrimidine ring with electron‐rich units that can act as electron donors, passivating Lewis acid defects.^[^ ^18^ ^]^ Second, BzMIM Br, as an imidazolium‐based ionic liquid, allows for the ionic radii of its cation to be tailored by selecting alkyl groups to meet the tolerance factor limit, thereby regulating the crystallization of perovskites and passivating defects.^[^ ^19^ ^]^ Consequently, a series of experiments were conducted to study the interactions between these materials and perovskite films.

**Figure 1:**
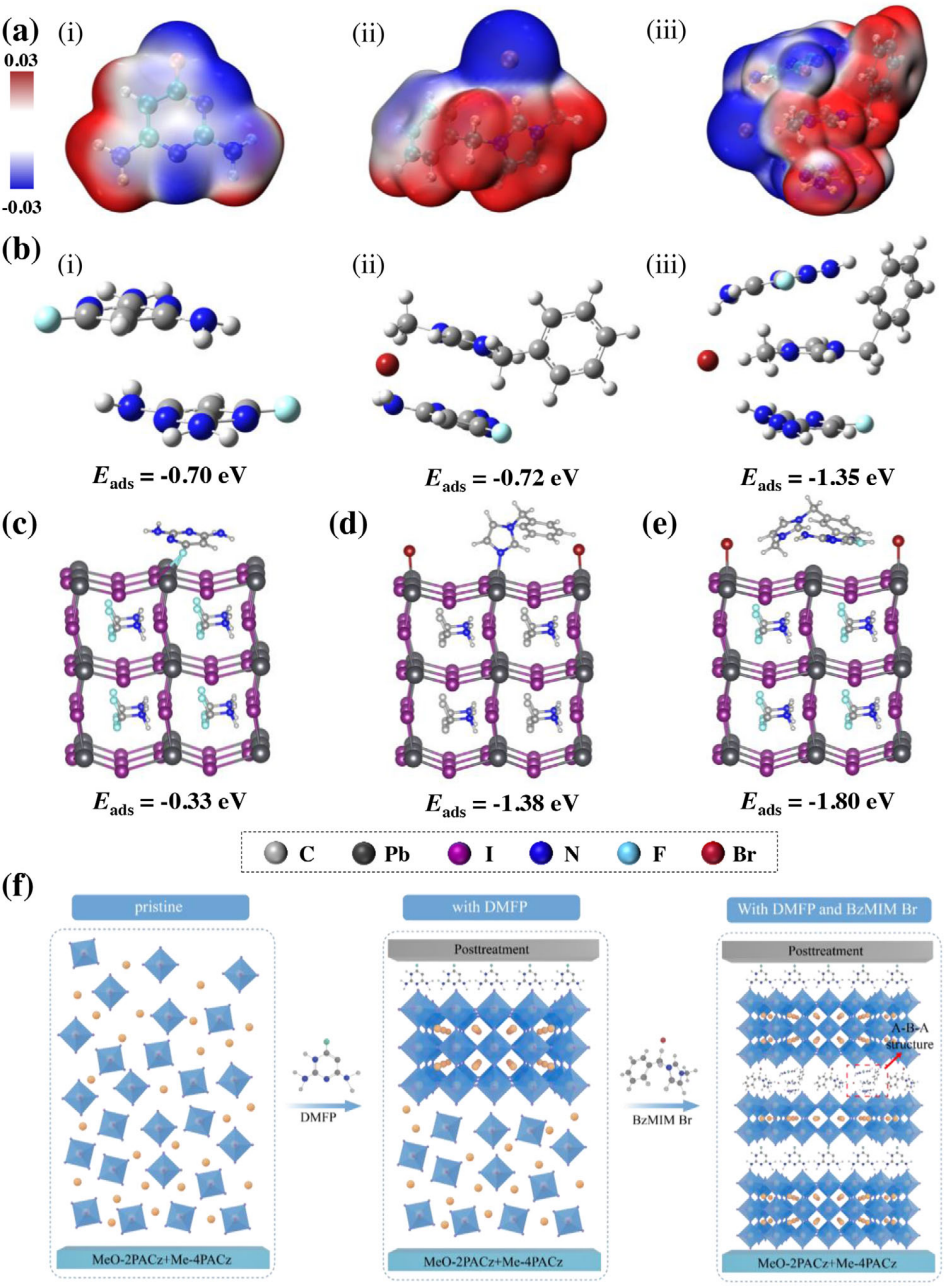
a) The electrostatic potential (ESP) of i) DMFP, ii) BzMIM Br, iii) DMFP‐BzMIM Br‐DMFP (A‐B‐A). b) DFT models of the adsorption energies (Eads) of i) DMFP‐DMFP; ii) DMFP‐BzMIM Br; iii) DMFP‐BzMIM Br‐DMFP. Schematic diagram of the interaction between the MAPbI_3_ perovskite and c) DMFP, d) BzMIM Br, and e) DMFP + BzMIM Br. f) Mechanism diagram of pristine, with DMFP, with DMFP and BzMIM Br‐treated at different positions within the devices.

**Figure** **1**a (i) illustrates the electrostatic potential (ESP) map of DMFP, showing the pyrimidine ring with increased electron density (blue), while the region above the amino groups exhibits low electron density (red). Notably, DMFP units are capable of forming Lewis acid‐base interactions with perovskite films through their electron‐rich aromatic moieties. Furthermore, the highly electronegative fluorine atom can reduce surface tension and form strong hydrogen bonds with A‐site cations, effectively blocking water molecule infiltration and significantly enhancing the stability of the perovskite film.^[^ ^20^ ^]^ Figure 1a (ii) shows the ESP of BzMIM Br, characterized by an overall positive ESP surface, with the bromine group exhibiting strong electronegativity, which plays a critical role in regulating the morphology and crystallization of perovskites.^[^ ^21^ ^]^ Figure 1a (iii) represents the ESP of A‐B‐A (DMFP‐BzMIM Br‐DMFP). In comparison to the ESP distribution of A‐B (DMFP‐BzMIM Br) in Figure S1b (Supporting Information), the ESP distribution of A‐B‐A shows a uniform distribution of positive and negative potentials in the vertical direction, whereas A‐B exhibits an alternating distribution of positive and negative potentials. Previous studies have reported the presence of positively and negatively charged defects within perovskites. In the horizontal direction, a uniform potential distribution can simultaneously passivate positively and negatively charged defects in perovskite films, reducing recombination centers, and improving the crystallization of perovskite films. In contrast, although an alternating potential distribution can interact more strongly with individual charged defects, it leads to higher defect density and poorer crystallinity.^[^ ^22^ ^]^ Therefore, this A‐B‐A “sandwich structure” doping passivation strategy establishes a uniform potential distribution in the vertical direction, effectively passivating charged defects, enhancing the crystalline orientation of perovskite grains, and significantly improving device performance and stability.

To demonstrate the existence of the A‐B‐A structure and its enhanced interaction with perovskite films, the adsorption energies (Eads) of A‐A (DMFP‐DMFP), A‐B, and A‐B‐A structures were calculated using Density Functional Theory (DFT), as shown in Figure 1b. The results indicate that all three adsorption energies are negative, signifying an exothermic process where energy is released as the adsorbate group transfers to the adsorbent surface, with values of −0.70, −0.72, and −1.35 eV, respectively. The absolute value of the adsorption energy for the A‐B‐A structure is the largest, indicating a stronger interaction between the adsorbate group and the adsorbent surface, making the A‐B‐A structure more stable and effective in passivating defects, consistent with the analysis results from the ESP diagram.^[^ ^23^ ^]^

To evaluate the effective role of the A‐B‐A structure in WBG PSC devices, corresponding experimental schemes were designed and studied. As shown in Figure S2 (Supporting Information), to passivate defects between the perovskite active layer and the electron transport layer, and enhance charge carrier extraction and transport rates, a scheme named Target 1 involved spin‐coating DMFP organic small molecules on the perovskite surface followed by annealing at 100 °C for 10 min. To suppress halide segregation and eliminate the influence of Pb vacancies on perovskite film crystallization, a scheme named Target 2 was implemented by adding the ionic liquid BzMIM Br to the perovskite precursor solution, spin‐coating it on the hole transport layer surface, and annealing at 100 °C for 30 min to obtain a black perovskite phase. This work combines compositional regulation and passivation engineering strategies by incorporating BzMIM Br into the perovskite precursor solution and annealing at 100 °C for 30 min while spin‐coating DMFP as a passivation layer on the perovskite film surface followed by annealing at 100 °C for 10 min (named Target 1 + Target 2). Experimental results demonstrate that through their synergistic action, BzMIM Br forms an active intermediate with the perovskite precursor solution, effectively delaying perovskite crystal growth. The slower crystal growth leads to larger perovskite grain sizes, reduced defect density, and significantly decreased lattice stress.^[^ ^24^ ^]^ Furthermore, the pyrimidine ring in the DMFP small organic molecules interacts with the perovskite film through Lewis acid‐base reactions, passivating defects, and utilizing the polar hydrophobicity of the F group, greatly enhancing stability.^[^ ^25^ ^]^ Additionally, based on the DFT calculations, the A‐B‐A structure formed after their synergistic action exhibits a uniform potential distribution, capable of simultaneously passivating positively and negatively charged defects, improving crystallinity, as evidenced by the larger grain sizes and vertical orientation observed in the experimental results.

To investigate how these two molecules influence WBG PSCs, DFT calculations were performed to explore the passivation abilities of DMFP, BzMIM Br, and their synergistic interactions with the surface acceptor defects of MAPbI_3_ perovskite (Figure 1c,e). Figure 1c reveals that DMFP interacts with MAPbI_3_ by forming a Pb‐F‐I covalent bond, attributed to the higher electronegativity of F compared to Br and I. Figure 1d shows that BzMIM Br interacts with MAPbI_3_ through the imidazole N binding to Pb sites, forming a C─N─Pb covalent bond and Br also binding to Pb, leading to the binding of dual sites with Pb vacancy defects, stabilizing the perovskite structure in the vertical direction. Figure 1e demonstrates the interaction of DMFP and BzMIM Br in synergy with MAPbI_3_, showing that DMFP is positioned at the bottom of BzMIM Br, with an E_ads_ value of −1.80 eV. This value is larger compared to the binding of individual molecules with MAPbI_3_, indicating that through the synergistic action of the two molecules, multisite binding with the perovskite occurs, inducing DMFP to adsorb at the bottom of BzMIM Br, forming an A‐B‐A structure for enhanced stability, thus validating the proposed hypothesis. Under the influence of the A‐B‐A structure, the perovskite film achieves larger grain sizes, superior crystalline orientation in the vertical direction, and enhanced charge carrier extraction and transport rates, effectively improving stability.^[^ ^26^ ^]^

To further validate the DFT calculations, Fourier transform infrared spectroscopy (FTIR) and X‐ray photoelectron spectroscopy (XPS) were employed to investigate the interaction mechanisms of various functional groups with perovskite. Figures S3 and S4 (Supporting Information) reveal that the introduction of BzMIM Br resulted in characteristic stretching vibration peaks of C─N in the 900–700 cm^−1^ range. With the addition of DMFP, the peak at 816 cm^−^¹ shifted to 817 cm^−^¹, indicating a stronger interaction between the C─N group and uncoordinated Pb^2^⁺ ions due to the synergistic action of DMFP and BzMIM Br.^[^ ^27^ ^]^ Additionally, characteristic stretching vibrations of C ═ N and N─H appeared at 2275–2225 cm^−^¹ and 3500–3250 cm^−^¹, respectively. In the presence of both DMFP and BzMIM Br, these peaks shifted to higher wavenumbers (C ═ N from 2250 to 2252 cm^−^¹; N─H from 3384 to 3401 cm^−^¹), suggesting the formation of a C ═ N…Pb covalent bond due to the donation of lone pair electrons from the pyrimidine N to the empty orbitals of Pb^2^⁺.^[^ ^28^ ^]^ The interaction is further enhanced by the NH₂ group in DMFP forming a strong N─H…I hydrogen bond with free halide ions (e.g., I⁻).^[^ ^29^ ^]^ XPS spectroscopic analysis provides better evidence for these interactions. As depicted in Figure S4 (Supporting Information), upon the individual introduction of DMFP or BzMIM Br, the Pb 4f spectral peak shifted toward higher binding energies. Following their synergistic interaction, the Pb 4f spectral peak exhibited a greater shift toward higher binding energies. Regarding the N 1s spectral peak, after the introduction of DMFP alone, it shifted toward higher binding energies, whereas upon the sole introduction of BzMIM Br, the N 1s spectral peak shifted toward lower binding energies. This alignment with the results from ESP, which indicates that the pyrimidine N in DMFP acts as an electron‐withdrawing group, thereby increasing electron density, while the imidazole N in BzMIM Br acts as an electron‐donating group, reducing the surrounding electron cloud density, and shifting toward higher binding energies.^[^ ^30^ ^]^ Furthermore, the movement of the I 3d spectral peak further illustrates the role of NH groups in DMFP, forming strong N─H…I hydrogen bonds. These variations in binding energies confirm the interaction between DMFP and BzMIM Br in synergy with perovskite, consistent with the FTIR results, ultimately enhancing the quality of WBG perovskite films.

By combining the A‐B‐A structure with the interactions between multiple functional groups and perovskite, we have hypothesized the growth mechanism of perovskite films. As shown in Figure 1f, the original perovskite film exhibits an irregular distribution during crystal growth due to the presence of numerous defects. Upon the introduction of DMFP, the electron‐rich environment of the pyrimidine ring coordinates with free Pb^2^⁺ ions, forming conjugated ligands on the surface, which leads to more regular crystal growth and improved film quality. However, due to surface effects, the defect density remains high in the internal and bottom regions of the perovskite crystals. Therefore, with the introduction of BzMIM Br, a synergistic effect is observed between the two molecules. BzMIM Br self‐induces DMFP to penetrate downward to the bottom of the perovskite, forming an A‐B‐A structure distributed throughout the entire perovskite.

The uniform potential distribution A‐B‐A structure can simultaneously passivate positive and negative charge defects. By utilizing the multi‐functional group interactions of DMFP and BzMIM Br, as depicted in Figure S5 (Supporting Information), BzMIM Br forms Br…Pb and C─N…Pb covalent bonds with the perovskite, constructing a molecular bridge in the vertical direction to reduce resistance during crystal growth, facilitating charge carrier transport. The bonding interactions of the pyrimidine N and NH groups in DMFP contribute to the formation of an A‐B‐A step sandwich structure, tightly connecting the entire perovskite in the vertical direction, allowing the crystals to preferentially grow downward vertically, thereby inhibiting vertical halide phase segregation.

**Figure 2:**
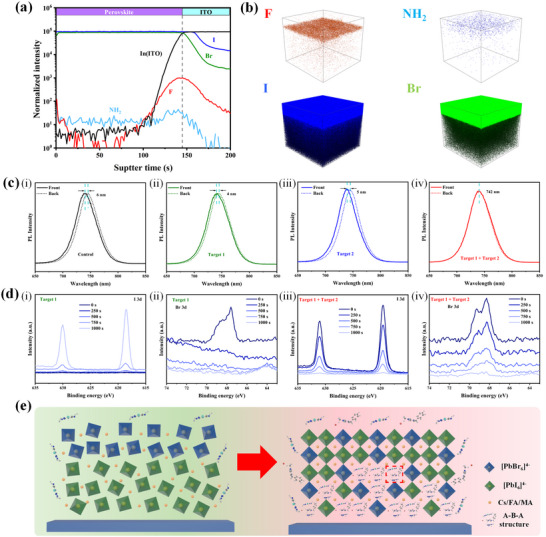
a) ToF‐SIMS depth‐profile negative ions analysis of perovskite film with DMFP and BzMIM Br collaborative‐treated. b) Reconstructed elemental 3D maps of F, NH, I, and Br ions traced in the depth profile for DMFP and BzMIM Br collaborative‐treated perovskite films. c) PL spectra of different post‐treatment perovskite/ITO excited from the perovskite surface (front) and the glass substrate (back). d) Depth profiling XPS spectra of I 3d for i) Target 1, iii) Target 1 + Target 2 and Br 3d for ii) Target 1, iv) Target 1 + Target 2 treated perovskite films with etching time. e) Br/I distribution images for Target 1 and Target 1 + Target 2 film, and Mechanism diagram of A‐B‐A structure in perovskite.

To validate the proposed growth mechanism, a series of characterizations were conducted. Firstly, we utilized DFT calculations to determine the binding energies of DMFP@MeO‐2PACz: Me‐4PACz, DMFP@Perovskite, and DMFP@PC_61_BM, as shown in Figure S6 (Supporting Information). The results indicate that E_b_ (DMFP@MeO‐2PACZ: Me‐4PACz) > E_b_ (DMFP@PC_61_BM) > E_b_ (DMFP@Perovskite), suggesting that DMFP exhibits a higher affinity for binding with the mixed hole transport layer and that the binding between them is more stable. This confirms that DMFP can distribute on the surface of the perovskite and self‐induce penetration to the bottom of the perovskite layer, forming an A‐B‐A structure in conjunction with BzMIM Br, thereby achieving a stepwise modulation effect on the perovskite. Next, time‐of‐flight secondary ion mass spectrometry (ToF‐SIMS) was employed to further investigate the distribution of DMFP in the perovskite film. As shown in **Figure** **2**a, the characteristic groups present in DMFP are F and NH_2_, primarily distributed on the surface and bottom of the perovskite film. In Figure S7 (Supporting Information), a uniform distribution of Pb within the perovskite can be observed, with a gradient downward distribution of A‐site cations (Cs, FA, MA), indicating the correspondence between the distribution of NH_2_ groups and DMFP. Figure 2b displays the corresponding 3D distribution image, demonstrating the uniform distribution of Br and I within the perovskite.^[^ ^31^ ^]^ In WBG PSCs, halide phase segregation, where Br and I exhibit non‐uniform distribution, is known to occur due to differences in the solubility and evaporation rates of PbBr_2_ and PbI_2_, leading to the accumulation of Br on the perovskite surface. This segregation results in regions rich in Br with high trap densities and deep energy level defects, while I‐rich regions can act as charge recombination centers, hindering carrier transport and causing non‐radiative recombination and significant V_oc_ losses. However, the controlled crystal growth in the vertical direction by the A‐B‐A structure inhibits halide segregation, eliminating the narrow‐bandgap I‐rich regions acting as recombination centers and the WBG Br‐rich regions with deep defect levels, effectively reducing unnecessary energy losses due to bandgap mismatches.^[^ ^32^ ^]^

To confirm the improvement in halide phase segregation, photoluminescence (PL) testing was conducted on the front and back surfaces of the perovskite films on glass substrates using an ITO/perovskite structure. As shown in Figure 2c for i) Control, ii) Target 1, iii) Target 2, and iv) Target 1 + Target 2 films, the presence of halide phase segregation in Control, Target 1, and Target 2 films led to differences in bandgaps between the front and back, manifested as peak position shifts in PL spectra. In the Target 1 + Target 2 film, no peak shifts were observed, indicating inhibition of halide segregation and there is no energy bandgap mismatch due to the A‐B‐A structure. Furthermore, we investigated the evolution of the thin film PL peak position under constant irradiation of a 475 nm laser, as shown in Figure S8 (Supporting Information). The PL peak position of the Control film exhibited a noticeable red shift after 5 min of illumination, shifting by 7 nm. The Target 1 film shifted by 6 nm, and the Target 2 film shifted by 4 nm. In contrast, the Target 1+Target 2 perovskite film showed a stable PL spectrum without any PL shift. This indicates that under the synergistic action of DMFP and BzMIM Br, effective inhibition of the wide‐bandgap perovskite phase segregation phenomenon occurred. This was achieved by filling halide vacancies to achieve compositional homogenization control, thereby suppressing phase segregation and enhancing the stability of the perovskite. Deep‐etching XPS measurements in Figure 2d further confirmed the uniform distribution of halides in the vertical direction. In the Target 1 film, the I 3d peak intensity increased with etching time, reaching its highest intensity at 1000 s, while Br distribution (Figure 2d (ii)) showed the opposite trend, indicating Br primarily located at the top of the perovskite and I at the bottom, exhibiting Br/I phase separation, as shown in Figure 2e.

For the Target 1 + Target 2 film, the I 3d peak intensity decreased in a step‐wise manner with etching time, similar to the trend observed for Br 3d (Figure 2d (ii,iii)), indicating effective mitigation of halide segregation after treatment with the A‐B‐A structure. The Br and I contents exhibited a step‐wise uniform distribution in the vertical direction, as shown in Figure 2e. Due to the equivalent Br/I ratios at the surface and bottom, there is no bandgap difference, consistent with the PL results. This suggests that the A‐B‐A structure, through its synergistic effect, occupies vacancies within the perovskite lattice, while the elements exhibit a step‐like distribution, the overall composition remains uniform after the vacancies are filled, favorable for enhancing the crystallinity of perovskite crystals, thereby inhibiting halide segregation, reduces recombination centers, lowers defect densities.^[^ ^33^ ^]^

**Figure 3:**
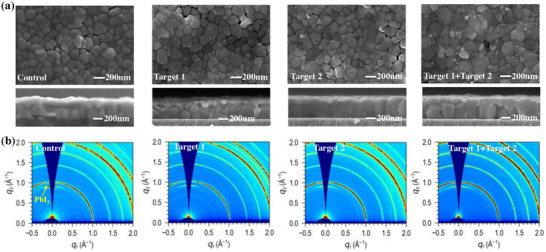
Characterizations of the Control, Target 1, Target 2, Target 1 + Target 2 films surface with different post‐treatments. a) Top‐view and cross‐sectional SEM images. b) GIWAXS patterns with an incidence angle of 0.2°.

We subsequently investigated the effects of DMFP and BzMIM Br on the crystallinity of perovskite films using cross‐sectional scanning electron microscopy (SEM), x‐ray diffraction (XRD), and atomic force microscopy (AFM) techniques. As shown in **Figure** **3**a, surface SEM images reveal that the grains in the Control film are disordered, while those in the Target 1 and Target 2 films show some improvement but still exhibit grain boundary defects that hinder charge carrier transport. In contrast, the grains in the Target 1 + Target 2 film are denser and larger, with average grain sizes of 118, 145, 145, and 220 nm respectively, as indicated in Figure S9 (Supporting Information). Denser and larger grains reduce grain boundaries and suppress defect‐induced nonradiative recombination in the films.^[^ ^34^ ^]^ AFM images (Figure S10, Supporting Information) demonstrate that the root mean square (RMS) roughness values for the Control, Target 1, Target 2, and Target 1 + Target 2 films are 24.2, 17.3, 17.8, and 16.7 nm, respectively. The lower roughness value of the Target 1 + Target 2 film indicates a smoother surface, promoting a denser connection between the perovskite film and the electron transport layer, thereby enhancing electron extraction efficiency. Moreover, cross‐sectional SEM images in Figure 3a reveal that the Target 1 + Target 2 film exhibits a single crystal grain in the vertical direction, whereas the Control film contains two layers of stacked grains arranged disorderly. The Target 1 film also shows stacked grains, and although some grains in the Target 2 film are single crystals, there are also stacked grains, with smaller grain sizes compared to the Target 1 + Target 2 film. It is known that the presence of grain boundaries increases resistance in the charge carrier transport process, thereby reducing device performance.^[^ ^35^ ^]^

Furthermore, the XRD patterns of perovskite films subjected to different post‐treatments were illustrated in Figure S11 (Supporting Information). The characteristic diffraction peaks of the (100) and (200) planes were observed at 14.2° and 28.4°, respectively, while the characteristic peak of PbI_2_ was detected at 12.8°. The results indicate the presence of PbI_2_ in the Control, Target 1, and Target 2 films, with the highest intensity in the Control film. In contrast, the corresponding PbI_2_ peak intensity was nearly absent in the Target 1 + Target 2 film, suggesting that under the influence of the A‐B‐A structure, coordination with free Pb^2+^ ions reduces PbI_2_ overflow, enhancing film stability. Compared to the Control, Target 1, and Target 2 films, the Target 1 + Target 2 film exhibits higher intensity characteristic peaks of the (100) and (200) planes, aligning with the SEM results mentioned earlier, indicating superior crystal face orientation that promotes the growth of larger grains, enhances film crystallinity, and facilitates efficient charge carrier extraction and transport.

Grazing‐incidence wide‐angle X‐ray scattering (GIWAXS) measurements were performed to investigate the crystalline differences in the perovskite films. At incidence angles of 0.2° and 0.5°, as shown in Figure 3b, the Control film exhibits a prominent PbI_2_ phase and weaker (100) signal, which can be attributed to Br/I phase segregation, resulting in lower crystal quality. In contrast, the PbI_2_ phase in the Target 1 and Target 2 films is reduced, and the (100) phase intensity is somewhat enhanced. Notably, the Target 1 + Target 2 film displays a significantly stronger (100) phase intensity compared to the other films, indicating superior crystal face orientation and higher crystallinity across both incidence angles (Figure S12, Supporting Information). The azimuthally averaged GIWAXS profiles at various incidence angles depicted in Figure S13 (Supporting Information) reveal that both the Target 1 and Target 2 films exhibit significantly strong (100) phase and reduced full width at half maximum (FWHM) values in the (100) direction at 0.2° and 0.5° angles. These findings suggest that the formation of the A‐B‐A structure by DMFP with BzMIM Br suppresses halide phase segregation in the vertical direction, enhances crystal quality, exhibits good crystallinity, utilizes superior crystal face orientation enlarges grain sizes, and improves device performance.^[^ ^36^ ^]^

**Figure 4:**
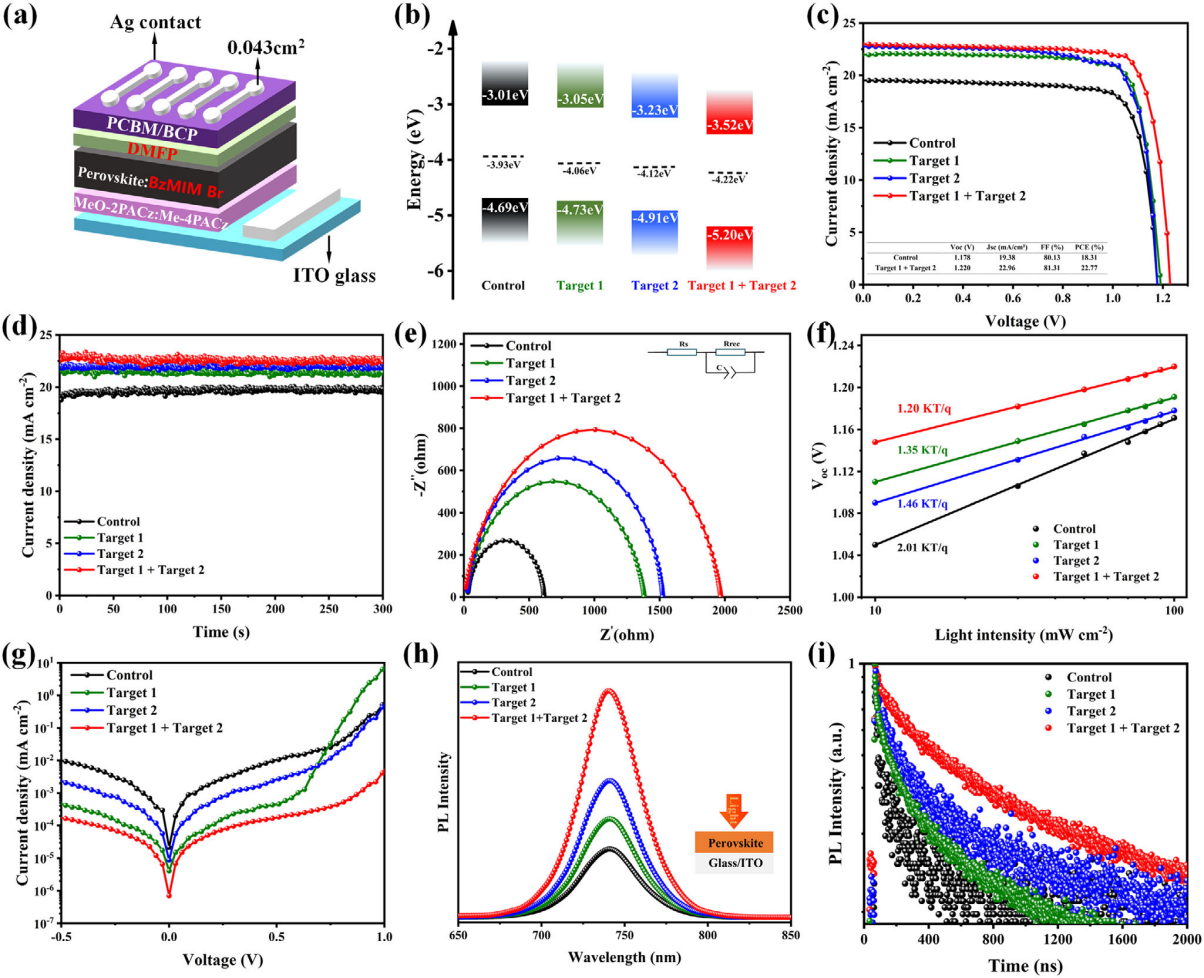
a) Device architecture of inverted WBG PSCs. b) Schematic energy‐level diagram of perovskite films with Control, Target 1, Target 2, Target 1 + Target 2. c) *J–V* curves, d) steady‐state power outputs, e) Nyquist plots at a bias of 1 V in the dark, f) Light‐intensity‐dependent V_oc_, and g) dark *J–V* curves of four types of PSCs. h) PL and i) TRPL spectra for Control, Target 1, Target 2, and Target 1 + Target 2 films excited from the perovskite side (top).

Then, we investigate the influence of the DMFP and BzMIM Br collaborative‐treated on device performance. The device structure is shown in **Figure** **4**a. The detailed preparation process can be found in the Experimental Section. Under the DMFP and BzMIM Br collaborative treatment, the energy level alignment of the perovskite was altered, as depicted in Figure 4b. The Control, Target 1, and Target 2 films all exhibited characteristics of p‐type semiconductors, with the Fermi level (E_F_) positioned closer to the valence band maximum (VB). In contrast, the Target 1 + Target 2 film demonstrated characteristics of an n‐type semiconductor, with E_F_ positioned closer to the conduction band minimum (CB). These were measured by UV photoelectron spectroscopy (UPS) (Figure S14, Supporting Information), Based on the bandgap (1.68 eV) and the positions of the VBM and the E_F_ of the perovskite film, the energy level alignment of perovskite films treated with different post‐processing steps was determined. The results indicate that the synergistic treatment of DMFP and BzMIM Br efficiently induces N‐type doping, leading to the formation of a P/N‐type heterojunction, enhancing the built‐in electric field, thus improving carrier transport and overall device efficiency.^[^ ^37^ ^]^

Moreover, we investigated the effect of different posttreatment on device performance, as shown in Figure 4c and Figure S15 (Supporting Information). It was discovered that the PSCs with DMFP and BzMIM Br collaborative‐treated presented the best photovoltaic performance in contrast with the control, Target 1, Target 2 device. The results show that the V_oc_ of devices was increased and the J_sc_ was significantly enhanced with the DMFP and BzMIM Br collaborative‐treated, this is due to the respective advantages of DMFP and BzMIM Br. Table S1 (Supporting Information) provides detailed photovoltaic parameters. It can be observed that after the DMFP and BzMIM Br collaborative‐treated, compared to pure BzMIM Br, the V_oc_ increased from 1.178 to 1.220 V, and compared to pure DMFP, the J_sc_ increased from 22.00 to 22.96 mA cm^−2^, and the final PCE is increased to 22.77%. Figure S16 (Supporting Information) presents the reverse and forward scanning curves of the DMFP and BzMIM Br collaborative‐treated device. The hysteresis factor was calculated using the following formula (Table S1, Supporting Information).

(1) $$HI=\frac{PCE_{Reverse}-PCE_{Forward}}{PCE_{Reverse}}$$

The HI values of Control, Target 1, Target 2, Target 1 + Target 2 devices were 2.4%, 1.7%, 1.5%, and 1%, indicating that the device by DMFP and BzMIM Br collaborative‐treated had negligible hysteresis, which might be attributed to the effective inhibition of ion migration by hydrogen bonding.^[^ ^38^ ^]^ Figure S17 (Supporting Information) displays the external quantum efficiency (EQE) spectra of the control, Target 1, Target 2, Target 1 + Target 2 devices. The integrated current densities were consistent with those extracted from the *J*–*V* curves. A stabilized PCE of 22.77% with a J_sc_ 22.96 mA cm^−2^ is obtained for the DMFP and BzMIM Br collaborative‐treated device when biased at 1.04 V, whereas that of the control device is only 18.31% with a J_sc_ is 19.38 mA cm^−2^ when biased at 1.02 V, the Target 1 device is only 22.00 mA cm^−2^ when biased at 1.02 V, and the Target 2 device is only 22.48 mA cm^−2^ when biased at 1.02 V. Combined with the results above we believe the J‐V measurement is reliable enough. Based on this, we prepared a 1 cm^2^ device via spin‐coating, and the *J*–*V* curve is shown in Figure S18 (Supporting Information) and detailed photovoltaic parameters are listed in Table S2 (Supporting Information). The results show that the application of different post‐treatments to the perovskite films in devices with an effective area of 1 cm^2^ follows corresponding patterns. Under the synergistic effect of DMFP and BzMIM Br, both J_sc_ and V_oc_ exhibit significant enhancements. The detailed parameters are as follows: the PCE is 18.54%, the V_oc_ is 1.188 V, the J_sc_ is 19.55 mA cm^−2^, and the FF is 79.81%. The electrical impedance spectroscopy (EIS) is measured in the dark to investigate the charge transfer dynamics of PSCs. In general, the arc at high frequencies is related to the series resistance (R_s_). While the incomplete semicircle at low frequency is generally assigned to the charge recombination resistance (R_rec_). As shown in Figure 4e and Table S3 (Supporting Information), the R_s_ values for Control, Target 1, Target 2, and Target 1 + Target 2 devices were 101.40, 77.21, 66.13, and 2.38 Ω, respectively. Meanwhile, R_rec_ values were 584.6, 1372, 1501, and 1967 Ω, respectively. The low R_s_ and high R_rec_ in the Target 1 + Target 2 devices, further indicate that the nonradiative recombination is reduced and the carrier transport capability is enhanced.^[^ ^39^ ^]^

To reveal the mechanism of the improved performance of WBG PSC devices, we performed a series of measurements. Light intensity‐dependent V_oc_ measurement is shown in Figure 4f, the V_oc_ versus the seminatural logarithm of light intensity presents a linear relationship. In principle, the slope depicted in the graph is related to the ideality factor (n). The results indicate that compared to the Control, Target 1, and Target 2 devices, the ideality factor (n) of the Target 1 + Target 2 device is 1.20, which is the smallest among the four devices. This suggests that the synergistic interaction of DMFP and BzMIM Br effectively suppresses trap‐assisted non‐radiative recombination.^[^ ^40^ ^]^ Additionally, we can demonstrate the reduction of non‐radiative recombination through dark J‐V testing. As shown in Figure 4h, the dark saturation current density (J_o_) can be extracted from the graph, following the formula:

(2) $$V_{oc}=\frac{nk_{B}T}{q}\ln\left(\frac{J_{t}}{J_{o}}+1\right)$$

where n, k_B_, q, T, J_t_, and J_o_ are the ideality factor, Boltzmann constant, elementary charge, absolute temperature, Theoretical current density, and reverse saturation current density under dark conditions, respectively. The results show that the device with the lowest J_o_ after the synergistic treatment with DMFP and BzMIM Br indicates a reduction in non‐radiative recombination caused by defects.^[^ ^41^ ^]^

To further validate the decrease in defect density, we conducted photoluminescence (PL) and time‐resolved photoluminescence (TRPL) measurements. The UV/visible absorption spectra of perovskite films subjected to different post‐treatments are shown in Figure S19 (Supporting Information). The results indicate that in the 500–800 nm range, the absorption spectra overlap, suggesting that the various post‐treatments did not alter the bandgap of the perovskite films, as confirmed by the corresponding Tauc plot showing a wide bandgap of 1.68 eV. The PL test results depicted in Figure 4h show that the PL intensity of both Target 1 and Target 2 films is relatively higher compared to the Control film, suggesting an enhanced carrier extraction capability after the interaction of DMFP and BzMIM Br. In contrast, the Target 1 + Target 2 film demonstrates enhanced PL intensity, with the highest intensity, suggesting a reduction in non‐radiative recombination and defect density after the synergistic action of DMFP and BzMIM Br. The peak positions of the PL spectra for the four films show no shift, indicating no change in the bandgap, consistent with the Tauc plot results mentioned earlier. The TRPL technique reveals the charge carrier transport dynamics of the different films, as illustrated in Figure 4i, the corresponding double exponential fitting results are presented in Table S4 (Supporting Information). The fitting data is calculated according to the following equation:

(3) $$f\left(t\right)=A_{1}\exp\left(\frac{-t}{\tau_{1}}\right)+A_{2}\exp\left(\frac{-t}{\tau_{2}}\right)+B$$

where average lifetime $\;\tau_{ave}=\frac{\left(A_{1}\tau_{1}^{2}+A_{2}\tau_{2}^{2}\right)}{\left(A_{1}\tau_{1}+A_{2}\tau_{2}\right)}\;$. The τ_*ave*_ for the Control, Target 1, Target 2 and Target 1 + Target 2 films are 423.02, 459.65, 527.46 and 684.33 ns. The Target 1 + Target 2 film exhibits the strongest PL intensity and the longest carrier lifetime, indicating a more pronounced effect of the synergistic action of DMFP and BzMIM Br, leading to a significant reduction in defect density.^[^ ^42^ ^]^ To further understand the enhancement in device performance, trap states in the perovskite were investigated using the space charge limited current (SCLC) method, the schematic diagram of the electron and hole device structures is illustrated in Figure S20 (Supporting Information). The density of trap‐states can be calculated by Equation (3):

(4) $$N_{trap}=\frac{2\varepsilon_{r}\varepsilon_{o}}{eL^{2}}V_{TFL}$$

where *N*_trap_ is the density of trap‐states, *ε*_r_ is the vacuum permittivity, *ε*_0_ is the relative dielectric constant for the perovskite film, *e* is the electron charge, *L* is the thickness of the perovskite layer (≈450 nm), and *V*_TFL_ is the trap‐filled limit voltage. As shown in Figure S21 (Supporting Information), the specific values of N_trap_ are presented in Table S5 (Supporting Information). The results indicate that the Target 1 + Target 2 film has the lowest electron defect density and hole defect density. The significant reduction in defect density may be attributed to the coordination of DMFP and BzMIM Br with free Pb^2+^ ions, as well as the ability of NH_2_ groups to form strong hydrogen bonds with I vacancies, effectively inhibiting ion migration and improving device stability while minimizing hysteresis effects.^[^ ^43^ ^]^

**Figure 5:**
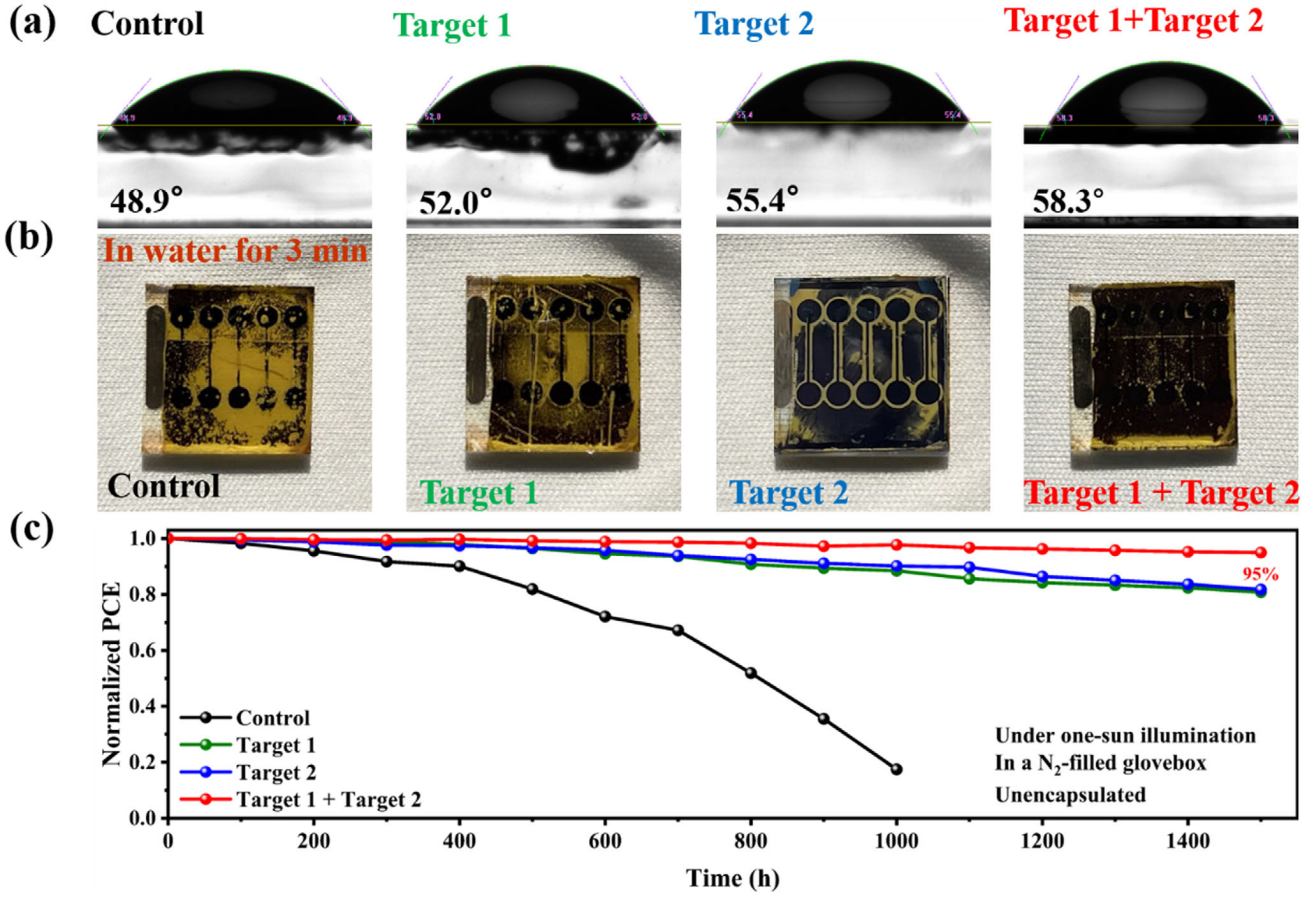
a) Water contact angles of different perovskite films. b) The photos of the morphology change of different perovskite films in water soak for 3 min under 25 °C. c) Long‐term stability measurements of unencapsulated devices under continuous light exposure and nitrogen atmosphere.

More importantly, the collaborative treatment with DMFP and BzMIM Br not only improved efficiency but also significantly enhanced the stability of the WBG PSCs. To further verify the stability enhancement, a series of studies were performed on unencapsulated films and devices. First, we investigated the effects of the treatment on the hydrophobicity of perovskite films using water contact angle measurements (**Figure** **5**a). The results show that the Target 1 + Target 2 film exhibited larger water contact angles compared to the Control, Target 1, and Target 2 films, indicating improved stability after the DMFP and BzMIM Br treatment.

Additionally, the enhancement in stability is further demonstrated by changes in the film morphology. As shown in Figure 5b, the Control, Target 1, and Target 2 films displayed significant degradation (turning yellow) after soaking in an aqueous solution for 3 min (in an environment without avoiding light, 25 ± 5 °C). In contrast, the Target 1 + Target 2 film maintained a stable morphology, suggesting that the F atom in DMFP forms strong ionic and intermolecular bonds with the perovskite, passivating the surface and reducing its sensitivity to further chemical reactions. Moreover, Br^−^ anions from BzMIM Br interact with Pb^2+^ ions and replace unstable I^−^ ions, thereby further enhancing the stability of the device. Meanwhile, the stability of devices based on Control, Target 1, Target 2, and Target 1 + Target 2 were measured under continuous light exposure and nitrogen atmosphere as shown in Figure 5c. Under unencapsulated and illumination under one‐sun equivalent conditions for over 1500 h, the PCE of the Control device had completely deteriorated by ≈1000 h. The Target 1 device based on DMFP maintained 80% of its original PCE, the Target 2 device based on BzMIM Br retained 81% of its initial PCE, while the Target 1 + Target 2 device, benefiting from the synergistic effect of DMFP and BzMIM Br, preserved 95% of its original PCE. The stabilized power output (SPO) of the Control, Target 1, Target 2, and Target 1 + Target 2 devices at the maximum power points (MPP) under constant AM 1.5G illumination for 300 s is measured to be 18.28%, 21.24%, 21.38%, and 22.72%, respectively, as shown in Figure S22 (Supporting Information), results indicate that the Control device exhibited a slight decrease. In contrast, the Target 1, Target 2, and Target 1+Target 2 devices demonstrated stable efficiency outputs, further confirming the enhancement in stability. The significant enhancement in device stability can be attributed to the collaborative formation of an A‐B‐A structure by both components, leading to strong interactions with the perovskite, resulting in a denser crystal structure that passivates defects at grain boundaries. This improvement not only enhances device performance but also greatly boosts stability.^[^ ^44^ ^]^

**Figure 6:**
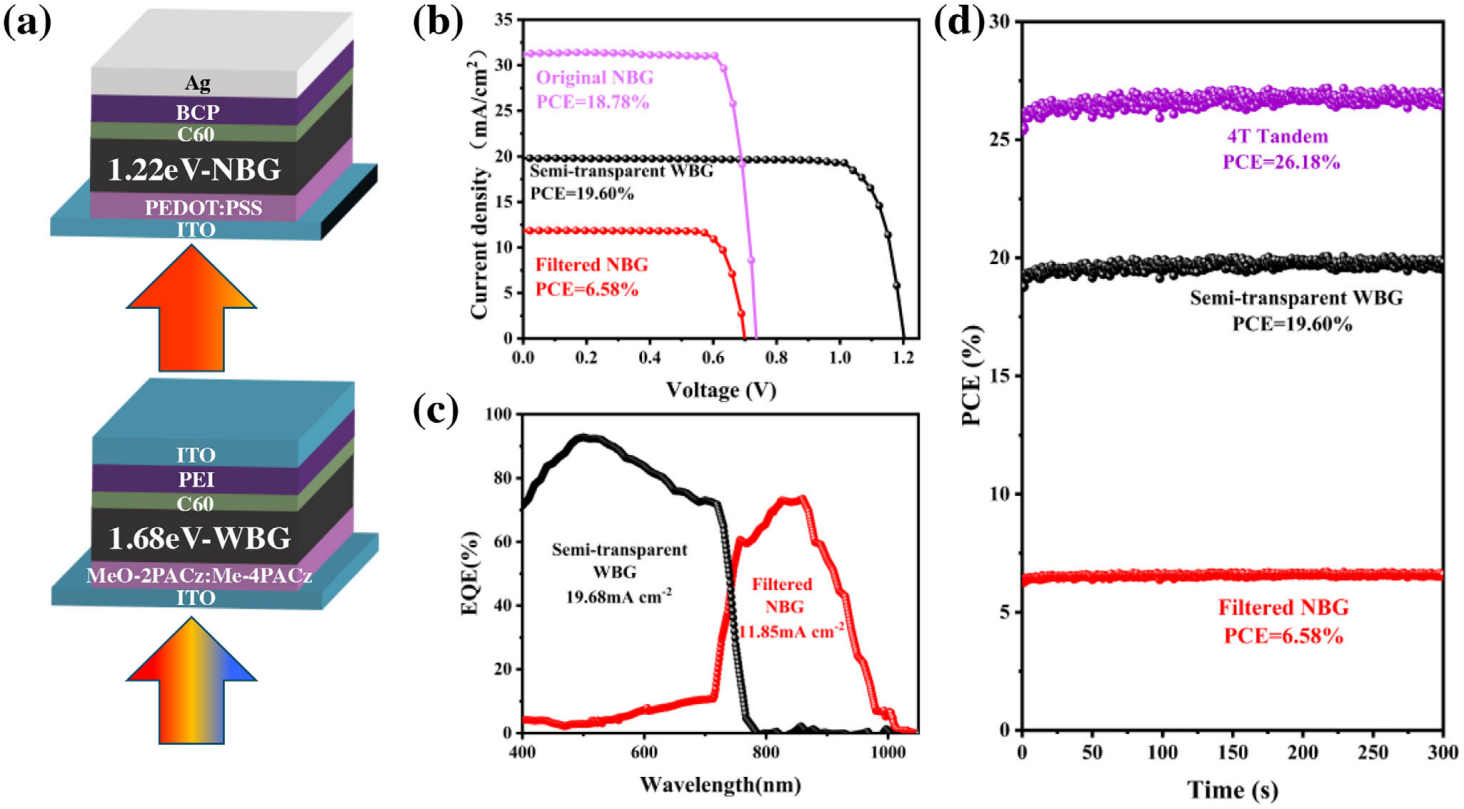
a) Devices structure of perovskite/perovskite 4T TSCs. b) *J*–*V* curves of a single‐junction semitransparent WBG device, an original single‐junction NBG device, and a filtered NBG device, respectively. c) EQE spectra of a single‐junction semitransparent WBG device and a filtered NBG device, respectively. d) Steady‐state power outputs were measured for a semitransparent WBG device and a filtered NBG device, respectively.

Building on the foundation of single‐junction perovskite solar cells, we fabricated 4T all‐perovskite tandem solar cells (TSCs). The structure of these TSCs consists of WBG PSCs with DMFP and BzMIM Br collaborative treatment as the top cell and NBG PSCs as the bottom cell (**Figure** **6**a). Remarkably, the semi‐transparent WBG PSC achieved a high PCE of 19.60%, with a V_oc_ of 1.203 V, a J_sc_ of 19.72 mA cm^−2^, and a FF of 82.44% (Figure 6b; Table S6, Supporting Information). The integrated J_sc_ from the EQE spectrum of the semi‐transparent device was 19.68% mA cm^−2^ (Figure 6c), which aligned well with the J‐V results shown in Figure 6b. The *J*–*V* curves and photovoltaic parameters of the original 1.22 eV bandgap mixed lead‐tin perovskite device are also shown in Figure 6b and Table S6 (Supporting Information). After filtering through the semi‐transparent WBG cell treated with DMFP and BzMIM Br, the bottom NBG cell achieved a PCE of 6.58% with a J_sc_ of 11.85 mA cm^−2^ under reverse scan. The integrated J_sc_ from the EQE spectrum of the NBG cell was also 11.85 mA cm^−2^ (Figure 6c), which matched well with the J‐V results. Figure 6d shows that the semi‐transparent WBG PSC and the filtered NBG PSC yielded steady‐state PCEs of 19.60% and 6.58%, respectively, under constant illumination at the MPP for 300 s. Ultimately, disregarding current matching, the stacked 4T all‐perovskite tandem device exhibited a remarkable PCE of 26.18% with a reverse voltage scan. These notable findings illustrate the effectiveness of integrating BzMIM Br as additives in the perovskite precursors and DMFP as passivation, facilitating the realization of high‐efficiency all‐perovskite TSCs.

## 3 Conclusion

In summary, we have demonstrated that through a compositional tuning strategy and passivation engineering synergy, the introduction of DMFP and BzMIM Br to interact strongly with the perovskite improves the performance and stability of WBG PSCs. DMFP self‐induces penetration to the bottom of the perovskite, forming a uniform potential distribution A‐B‐A sandwich structure with doped BzMIM Br, passivating both positive and negative charge defects, improving film crystallinity, and obtaining high‐quality perovskite films. Additionally, it effectively reduces defect density by inhibiting non‐radiative recombination caused by halide segregation. The results demonstrate that WBG PSCs treated with DMFP and BzMIM Br achieved an impressive PCE of 22.77%, a V_oc_ of 1.220 V, and an ultra‐high J_sc_ of 22.96 mA cm^−2^. The unencapsulated devices retained over 95% of initial efficiency after 1500 h of continuous illumination under one‐sun equivalent conditions in a nitrogen atmosphere. Subsequently, semi‐transparent devices were prepared with a PCE of 19.60%, coupled with Pb‐Sn perovskite‐based bottom devices with a 1.22 eV bandgap, achieving a PCE of 26.18% for a 4T all‐perovskite tandem solar cell. Furthermore, in terms of large‐area devices, devices with an area of 1 cm^2^ prepared via spin‐coating achieved a PCE of 18.54% after treatment with DMFP and BzMIM Br.

## Conflict of Interest

The authors declare no conflict of interest.

## Author Contributions

X.Y. conceived the idea and performed experiments and analysis; Y.Z., K.L., and X.C. helped with SEM, XRD, and UPS measurements; M.Z. and Z.X. helped with PL, GWIAXS, and FTIR measurements, and discussed some of the results. X.Y. and X.Z. helped with the fabrication of semitransparent WBG perovskite solar cells; Y.Z. helped with 4‐terminal all perovskite solar cells. X.Y. wrote the manuscript, and T.Z., H.W., W.M., and S.L. supervised this project and contributed to the manuscript review and editing. All authors discussed the results and commented on the manuscript.

## Supporting information



Supporting Information

## Data Availability

The data that support the findings of this study are available in the supplementary material of this article.
