# Supplementary material for: Self‐Induced A‐B‐A Structure Enables Efficient Wide‐Bandgap Perovskite Solar Cells and Tandems
Source: Adv Sci (Weinh). 2025 Mar 5;12(16):2413749. doi: 10.1002/advs.202413749 (PMC12021109; doi:10.1002/advs.202413749)
Supplement: Supplementary file 1 — Supporting Information [file ADVS-12-2413749-s001.docx]

**Supporting Information**

**Self-induced A-B-A Structure Enables Efficient Wide-Bandgap Perovskite Solar Cells and Tandems**

*Xixi Yu, Yong Zhu, Kunpeng Li, Xiong Chang, Mengni Zhou, Zhewen Xie, Xing Zhu, Hua Wang*, Wenhui Ma*, Shaoyuan Li*, Tao Zhu**

X Yu, Y Zhu, K Li, X Chang, M Zhou, Z Xie, X Zhu, H Wang, W Ma, S Li, T Zhu

Faculty of Metallurgical and Energy Engineering

Kunming University of Science and Technology

Kunming, 650093, P. R. China

E-mails: [zhutao3306@163.com](mailto:zhutao3306@163.com); lsy415808550@163.com

H Wang

State Key Laboratory of Complex Nonferrous Metal Resources Clean Utilization

Kunming University of Science and Technology

Kunming 650093, P. R. China

E-mails: [wanghua65@163.com](mailto:wanghua65@163.com)

W Ma

School of Engineering

Yunnan University

Kunming 650500, China

E-mails: [mawenhui@ynu.edu.cn](mailto:mawenhui@ynu.edu.cn)

T Zhu

Yunnan Key Laboratory of Clean Energy and Energy Storage Technology

Kunming 650093, P. R. China

**Experimental Section**

**Materials**

N, N-dimethylformamide (DMF, 99.8%), and dimethyl sulfoxide (DMSO, 99.9%) were purchased from Sigma-Aldrich. Isopropanol (IPA, 99.5%) were purchased from J&K. Lead iodide (PbI_2_, 99.999%), lead bromide (PbBr_2_, 99.99%), Cesium iodide (CsI, 99.99%), Methylammonium bromide (MABr, 99.99%), holeblocking material 2,9-dimethyl-4,7-diphenyl-1,10-phenanthroline (BCP, 99.5%) were purchased from Xi'an Yuri Solar Co., Ltd. Formamidinium iodide (H_2_N=CHNH_2_I; FAI), [6,6]-phenyl-C_61_-butyric acid methyl ester (PCBM, 99.9%), PEDOT: PSS (Clevious PVP AI4083) was purchased from Advanced Election Technology Co., Ltd. Fullerene (C60, 99%) is purchased from Vizuchem Co., Ltd. Polyethyleneimine (PEI) was purchased from Beijing Innochem Science & Technology Co., Ltd. Indium Tin Oxide (ITO coated glass, square resistance 15 Ω) is purchased from Advanced Election Technology Co., Acid (Meo-2PACZ),[4-(3,6-Dimethyl-9H-carbazol-9-yl)butyl]phosphonic Acid (Me-4PACZ) is purchased from TCI. Other materials were purchased from Alfa Aesar. All salts and solvents were used as received without any further purification.

**Wide-bandgap perovskite precursor preparation**

A 1.50 M Cs_0.05_(FA_0.77_MA_0.23_)_0.95_Pb(I_0.77_Br_0.23_)_3_ wide-bandgap perovskite precursor was prepared by adding 19.48 mg of CsI, 188.69 mg of FAI, 36.70 mg of MABr, 532.47 mg of PbI_2_, 126.62 mg of PbBr_2_ and with 2% PbX_2_ excess, respectively (X=I or Br) in an alloyed solvent of DMF and DMSO with a volume ratio of 4:1. Then, the solutions were stirred 60 min at 60℃ temperature in a N_2_ glovebox. For the perovskite with BzMIM Br additive, BzMIM Br was added at a volume ratio of 60:1 (perovskite: BzMIM Br). Before preparing perovskite films, the precursor solutions were filtered through a 0.45 mm polytetrafluoroethylene (PTFE) filter.

**Fabrication of single junction wide-bandgap perovskite device**

The ITO glass substrates were sequentially cleaned with cleanser, isopropanol, deionized water, and ethyl alcohol in a sonication bath for 15 min. Then we treated the dried substrates with UV Ozone for 30 min before use. For using mixed-solution MeO-2PACz and Me-4PACz as hole transportation layer, the mixed-solution in ethanol with a concentration of 0.5 mg/ml, volume ratio 3:1 was spin-coated at 5000 rpm for 30 s and annealed at 100℃ for 10 min. For the WBG perovskites, 50 μL perovskite solution was uniformly dropped on the HTL by spin-coated at 1100 rpm for 2 s, and 6000 rpm for 35 s; In the last 10 s of spin coating, 150 μL of CB was dropped on the spinning substrates. Then, the perovskite film was immediately placed on a hot plate and annealed at 100℃ for 30 min. For the target devices, the solution of DMFP in isopropanol with an optimized concentration of 1 mg/ml was dropped on the room-temperature perovskite films and dynamically spin-coated at a speed of 4000 rpm for 30 s. Then, PCBM (20mg/ml in chlorobenzene) and BCP (saturated ethanol solution) were spin-coated with spinning speeds of 1500 rpm for 50 s and 6000 rpm for 25 s, respectively. Lastly, 120 nm Ag was evaporated under a high vacuum (＜8×10^-4^ Pa) on the substrates to form electrodes.

**Fabrication of semi-transparent WBG PSCs**

For semi-transparent devices, C60 with a thickness of 20 nm was used to replace PCBM. Then, 1 mg/ml PEI solution (dissolved in IPA) was spin-coated on the top of the C60 layer at 5000 rpm for 30 s. And then, 120 nm ITO was sputtered at a power of 60W under Ar pressure of 3mTorr, Finally, the 100 nm Ag finger was deposited on the edge of the active area of the ITO electrode.

**Fabrication of narrow-bandgap (NBG) PSCs**

The ITO glass substrates were sequentially cleaned with cleanser, isopropanol, deionized water, and ethyl alcohol in a sonication bath for 15 min. Then we treated the dried substrates with UV Ozone for 30 min before use. Then, PEDOT: PSS was spin-coated onto the ITO substrate at 4500 rpm for 30 s and annealed at 140 ℃ for 20 min in ambient air. In the following, the FA_0.7_MA_0.3_Pb_0.5_Sn_0.5_I_3_-based precursors were spin-coated onto the substrates in an N_2_-filled glovebox at 1000 rpm for 10 s and then at 4000 rpm for 40 s, and 300 μL of CB was dripped onto the spinning substrates at 30 s after the starting of the spin-coating followed by annealing at 100 ℃ for 10 min. In the end, C60 (20nm), BCP (7nm), and Ag (120nm) were sequentially deposited on the narrow-bandgap perovskite layers to complete solar cells by thermal evaporation under a high vacuum system (<5×10^-4^ Pa).

**Device characterization**

**Single Junction WBG Bandgap Solar Cells:** The crystalline structure of perovskite films was analyzed by an X-ray diffractometer (XRD, D8 Advance, Bruker). The absorption spectra of perovskite films and precursor solutions were performed using a spectrophotometer (UV-vis, mini UV-1208 model, Shimadzu). Incident photon to current efficiency (IPCE) measurements were recorded with a QE/IPCE system (Enli Technology Co. Ltd). Time-of-flight secondary ion mass spectra (ToF-SIMS) were detected by ION TOF ToF SIMS 5-100 (Primary ion beam: Bi^3+^, 30 keV, incident angle: 45 deg, scanning area: 150*150 um^2^, pixel: 128*128, beam current: 0.48 pA). EQE measurements for tandem devices were performed in ambient air. The steady-state photoluminescence (PL) and time-resolved photoluminescence (TRPL) spectra were measured using a Delta Flex fluorescence spectrometer (HORIBA). J-V characterizations and stabilized power outputs (SPO) of the PSCs were performed under air mass 1.5 global (AM 1.5G) conditions (Enli Technology Co. Ltd) in nitrogen. The J-V measurements were performed with a scan rate of 0.02 V s^-1^ ranging from 1.2 to -0.2 V and then reversed again from -0.2 to 1.2 V. The top-view and cross-sectional images of perovskite films and devices were observed with a field-emission scanning electron microscope (SEM, Zeiss SIGMA). The surface morphology status further was measured by tapping-mode atomic force microscopy (AFM, NanoScope NS3A system). X-ray photoelectron spectroscopy (XPS) spectra were measured using an XPS/UPS system (Thermo Scientific, Escalab 250Xi). Space charge limited current (SCLC) curves of single-carrier devices were measured with a scan rate of 20 mV/s ranging from 0 to 4 V using a Keithley 2400 source meter in the dark. Electrochemical impedance spectroscopy (EIS) spectra were observed on a Chenhua CHI660E electrochemical workstation under dark conditions. A small AC disturbed the voltage of 20 mV, a DC bias voltage of 1.0 V, and a frequency scope from 1 to 100 kHz were kept during the EIS measurements.

**Fourier-transform infrared (FTIR) measurements:** Fourier-transform infrared (FTIR) spectroscopy testing can analyze the interactions between molecules and PbI2. The measurements were conducted using a Thermo Scientific Nicolet iS20 instrument employing a liquid cell technique. In a dry environment, the liquid sample was gently dropped onto the surface of the ATR crystal, continuously adding liquid to ensure coverage of the ATR crystal surface by the liquid droplet. Subsequently, the sample spectrum was measured, and the acquired data was processed to obtain the final results.

**Four-terminal (4T) tandem solar cells:** For 4T TSCs measurements, PCEs of top semi-transparent WBG PSCs were first measured. According to the previous report, silicone oil was also employed as an interconnection layer in the air gap between the semi-transparent cells and the NBG cells to minimize optical loss. Subsequently, PCEs of bottom NBG PSCs were tested after being light-filtered by top semi-transparent WBG PSCs. The illumination area corresponds to the effective area of the semi-transparent perovskite solar cells. PCEs of 4T all-perovskite tandem solar cells were equal to the sum of the efficiencies of semi-transparent WBG and filtered NBG PSCs.

**DFT calculations**

**The adsorption energies:** the DFT calculations were performed using the Vienna Ab-initio Simulation Package (VASP)^[1]^. The Perdew-Burke-Ernzerhof (PBE) functional within the generalized gradient approximation (GGA) method was employed to account for the exchange-correlation effects^[2]^. The core-valence interactions were treated using the projected augmented wave (PAW) method^[3]^. A plane wave cutoff energy of 400 eV was applied in the calculations. Structural optimization was carried out with energy and force convergence criteria set to 1.0×10-4 eV and 0.05 eV Å-1, respectively. The Brillouin zone was sampled using a 1×1×1 K-point grid. Grimme’s DFT-D3 methodology^[4]^ was used to describe the dispersion interactions.

The adsorption energies (*E*_ads_) are calculated by

*E*_ads_ = *E*_*BzMIMBr_–*E*_BzMIMBr_–*E*_sub_

*E*_ads_ = *E*_*6-fluoropyrimidine-2-4-diamine_–*E*_6-fluoropyrimidine-2-4-diamine_–*E*_sub_

where *E*_BzMIMBr_ and *E*_*BzMIMBr_ represent the energies before and after the adsorption of BzMIMBr on the substrates, respectively. *E*_6-fluoropyrimidine-2-4-diamine_ and *E*_*6-fluoropyrimidine-2-4-diamine_ represent the energies before and after the adsorption of 6-fluoropyrimidine-2-4-diamine on the substrates. *E*_sub_ is the energy of MAPbI_3_ surface.

**The electrostatic potential plots:** All calculations in the electrostatic potential plots were performed using the Gaussian 09 program package. Full geometry optimizations were performed to locate all the stationary points, using the B3LYP with the 6-31G(d,p) basis. Dispersion corrections were computed with Grimme's D3 (BJ) method in optimization5. Frequency analysis was carried out to verify the optimized geometry to be a minimum transition structure and to obtain the thermal corrections for the Gibbs free energy. Harmonic vibrational frequency was performed at the same level to guarantee that there is no imaginary frequency in the molecules, i.e. they are located on the minima of potential energy surface. Convergence parameters of the default threshold were retained (maximum force within 4.5×10−4 Hartrees/Bohr and root mean square (RMS) force within 3.0×10−4 Hartrees/Radian) to obtain the optimized structure. The optimal structure was identified given that all calculations for structural optimization were successfully converged within the convergence threshold of no imaginary frequency, during the process of vibration analysis.

Fig. S1. a) Chemical structure and ball-and-stick model of DMFP and BzMIM Br. b) The electrostatic potential (ESP) of A-B (DMFP-BzMIM Br).

Fig. S2. Schematic illustration of the fabrication steps for the Target 1 (DMFP-treated), Target 2 (BzMIM Br-treated), and Target 1 + Target 2 (DMFP and BzMIM Br collaborative-treated) perovskite films.

Fig. S3. (a, b, and c) FTIR spectra of the PbI_2_, PbI_2_ /Target 1, PbI_2_ /Target 2, and PbI_2_ /Target 1 + Target 2.

Fig. S4. XPS spectra of a) Pb 4f, b) N 1s, and c) I 3d peaks of Control, Target 1, Target 2, and Target 1 + Target 2 films.

Fig. S5. The schematic diagram of the chemical interaction between A-B-A (DMFP-BzMIM Br-DMFP) and perovskite.

Fig. S6. Binding model between a) DMFP and Me0-2PACz: Me-4PACz, b) DMFP and perovskite, and c) DMFP and PC_61_BM, and Binding energy values.

Fig. S7. ToF-SIMS depth-profile positive ions analysis of perovskite film with DMFP and BzMIM Br collaborative-treated.

Fig. S8. Normalized PL spectra of a) control, b) Target 1, c) Target 2, and d) Target 1+Target 2 films under constant illumination of a 475 nm laser for 5 min.

Fig. S9. The statistics of grain sizes of a) Control, b) Target 1, c) Target 2, and d) Target 1 + Target 2 films.

Fig. S10. Top views AFM images of a) Control, b) Target 1, c) Target 2, and d) Target 1 + Target 2 films.

Fig. S11. XRD patterns of Control, Target 1, Target 2, and Target 1 + Target 2 films.

Fig. S12. GIWAXS patterns of a) Control, b) Target 1, c) Target 2, and d) Target 1 + Target 2 films with an incidence angle of 0.5°.

Fig. S13. Azimuthally averaged GIWAXS profiles of different post-treatment perovskite films collected at an incidence angle of a) 0.2°and b) 0.5°.

Fig. S14. UPS of the a) Corresponding cutoff (E_cut-of_) and b) Fermi level (E_F_) energy regions of Control, Target 1, Target 2, and Target 1 + Target 2 films.

Fig. S15. Statistics a) of V_oc_, b) J_sc_, c) of FF and d) of PCE for four types of devices.

Fig. S16. J-V characteristics curves under reverse and forward scanning.

Fig. S17. EQE spectra of single junction wide bandgap (WBG) devices, and the corresponding derivative *dQEQE e/dE* versus E and calculated Eip g value.


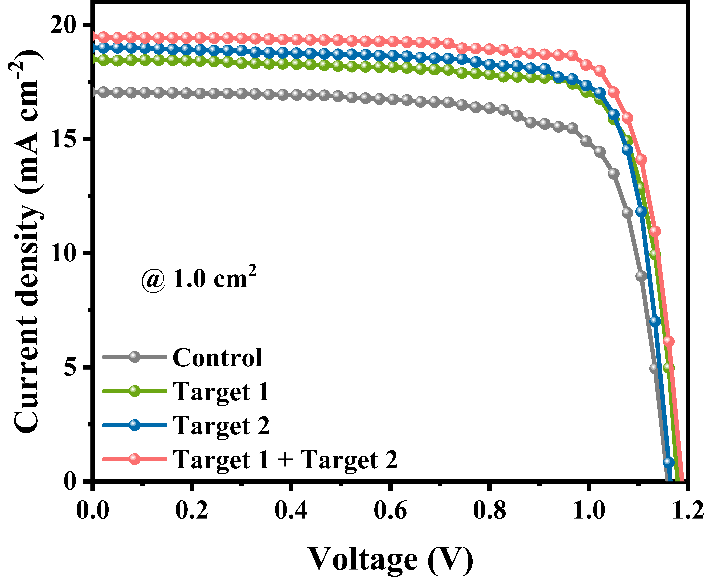


Fig. S18. *J-V* characteristics curves of 1.0 cm^2^ improved device with Control, Target 1, Target 2, and Target 1 + Target 2.

Fig. S19. The absorption spectra of the Control, Target 1, Target 2, and Target 1 + Target 2 films. The inset is the Tauc plots of the perovskite films.

Fig. S20. Device architecture of a) electron-only and b) hole-only devices.

Fig. S21. SCLC curves of the devices using Control, Target 1, Target 2, and Target 1+Target 2 films.


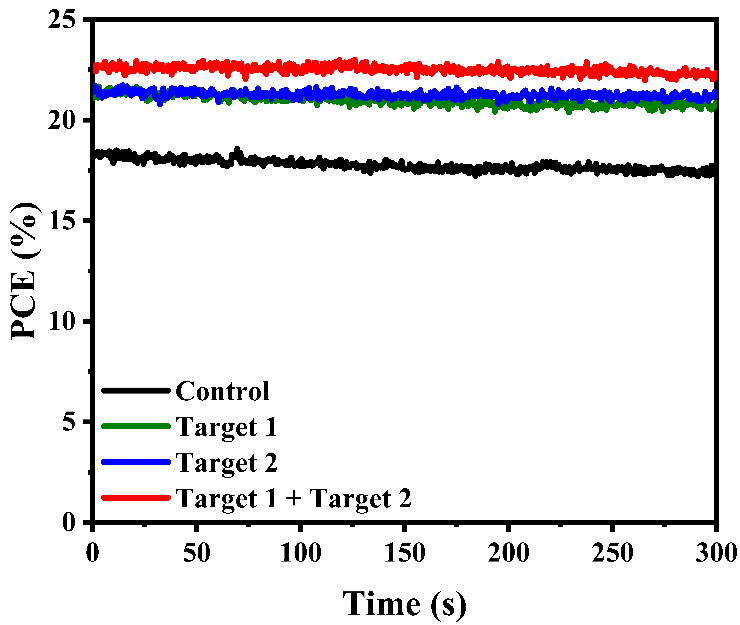


Fig. S22. Steady-state power outputs were measured for Control, Target 1, Target 2, and Target 1 + Target 2 devices, respectively.

Table S1. The photovoltaic parameters of different post-treatment devices with V_oc_, J_sc_, FF, and PCE (active area: 0.043 cm^2^).

| Sample | Scan  direction | Voc  (V) | FF  (%) | Jsc  (mA/cm^2^) | PCE  (%) | H  (%) |
| --- | --- | --- | --- | --- | --- | --- |
| Control | Reverse | 1.178 | 80.13 | 19.38 | 18.31 | 2.4 |
|  | Forward | 1.172 | 78.98 | 19.30 | 17.87 |  |
| Target 1 | Reverse | 1.191 | 81.18 | 22.00 | 21.25 | 1.7 |
|  | Forward | 1.192 | 80.79 | 21.68 | 20.89 |  |
| Target 2 | Reverse | 1.178 | 80.80 | 22.48 | 21.40 | 1.5 |
|  | Forward | 1.172 | 80.95 | 22.21 | 21.07 |  |
| Target 1 + Target 2 | Reverse | 1.220 | 81.31 | 22.96 | 22.77 | 1.0 |
|  | Forward | 1.227 | 81.12 | 22.64 | 22.53 |  |

Table S2. The photovoltaic parameters of different post-treatment devices with V_oc_, J_sc_, FF, and PCE (active area: 1.0 cm^2^).

| Device | Voc  (V) | FF  (%) | Jsc  (mA/cm^2^) | PCE  (%) |
| --- | --- | --- | --- | --- |
| Control | 1.159 | 76.52 | 17.27 | 15.32 |
| Target 1 | 1.179 | 78.83 | 18.46 | 17.16 |
| Target 2 | 1.164 | 79.12 | 19.00 | 17.50 |
| Target 1 +Target 2 | 1.188 | 79.81 | 19.55 | 18.54 |

Table S3. series resistance (R_s_) and the recombination resistance (R_rec_) obtained by EIS measurement.

| sample | R_S_(Ω) | R_­rec_(Ω) |
| --- | --- | --- |
| Control | 101.40 | 584.6 |
| Target 1 | 77.21 | 1372 |
| Target 2 | 66.13 | 1501 |
| Target 1 + Target 2 | 2.38 | 1967 |

Table S4. TRPL fitting results of WBG perovskite films.

| sample | τ_1_ (ns) | τ_2_ (ns) | τ_avg_ (ns) |
| --- | --- | --- | --- |
| Control | 38.45 | 521.66 | 423.02 |
| Target 1 | 75.30 | 534.28 | 459.65 |
| Target 2 | 97.19 | 613.17 | 527.46 |
| Target 1 + Target 2 | 114.92 | 722.01 | 684.33 |

Table S5. Defect density (N_t_) calculated by SCLC of WBG perovskites with Control, Target 1, Target 2, and Target 1 + Target 2 devices.

| **Sample** | **Structure** | **N_t_ (cm^-3^)** |
| --- | --- | --- |
| Control | Electron-only | 2.21×10^16^ |
|  | Hole-only | 4.81×10^16^ |
| Target 1 | Electron-only | 1.23×10^16^ |
|  | Hole-only | 4.10×10^16^ |
| Target 2 | Electron-only | 9.10×10^15^ |
|  | Hole-only | 3.58×10^16^ |
| Target 1 + Target 2 | Electron-only | 7.80×10^15^ |
|  | Hole-only | 3.51×10^16^ |

Table S6. Photovoltaic parameters of the optimized Wide-Eg semitransparent device, Low-Eg device, and 4T tandem device.

| Device | V_oc_  (V) | FF  (%) | J_sc_  (mA/cm2) | PCE  (%) |
| --- | --- | --- | --- | --- |
| 1.68 eV-semitransparent | 1.203 | 82.44 | 19.72 | 19.60 |
| 1.25 eV-NBG | 0.736 | 81.79 | 31.21 | 18.78 |
| 1.25 eV-filtered | 0.695 | 80.23 | 11.85 | 6.59 |
| 4T-TSC |  |  |  | 26.18 |

Table S7. Summary of 1.68 eV wide‐bandgap single‐junction perovskite solar cells reported in the literature.

| Perovskite | Year | Jsc  (mA m^-2^) | Voc  (V) | PCE  (%) | Ref |
| --- | --- | --- | --- | --- | --- |
| Cs_0.05_(FA_0.77_MA_0.23_)_0.95_Pb(I_0.77_Br_0.23_)_3_ | 2020 | 20.7 | 1.224 | 20.8 | ^[5]^ |
| Rb_0.05_Cs_0.05_(FA_0.75_MA_0.25_)Pb(I_0.75_Br_0.25_)_3_ | 2022 | 20.98 | 1.207 | 20.11 | ^[6]^ |
| Cs_0.22_FA_0.78_Pb(I_0.85_Br_0.15_)_3_ | 2022 | 20.65 | 1.200 | 19.52 | ^[7]^ |
| Cs_0.05_MA_0.15_FA_0.8_Pb(I_0.75_Br_0.25_)_3_ | 2023 | 20.94 | 1.200 | 20.38 | ^[8]^ |
| Cs_0.05_(FA_0.77_MA_0.23_)_0.95_Pb(I_0.77_Br_0.23_)_3_ | 2023 | 21.16 | 1.239 | 21.63 | ^[9]^ |
| Cs_0.05_FA_0.8_MA_0.15_Pb(I_0.76_Br_0.24_)_3_ | 2023 | 21.5 | 1.250 | 22.7 | ^[10]^ |
| FA_0.7_MA_0.05_Cs_0.25_Pb(I_0.8_Br_0.2_)_3_ | 2024 | 20.9 | 1.243 | 21.48 | ^[11]^ |
| FA_0.8_Cs_0.2_Pb(I_0.8_Br_0.2_)_3_ | 2024 | 21.48 | 1.220 | 21.72 | ^[12]^ |
| Cs_0.05_FA_0.80_MA_0.15_Pb(I_0.75_Br_0.25_)_3_ | 2024 | 20.88 | 1.265 | 22.3 | ^[13]^ |
| Cs_0.05_FA_0.8_MA_0.15_PbI_2.25_Br_0.75_ | 2024 | 22.18 | 1.216 | 22.35 | ^[14]^ |
| Cs_0.05_(FA_0.95_MA_0.05_)_0.95_PbI_3_ | 2024 | 21.28 | 1.290 | 22.92 | ^[15]^ |
| Cs_0.05_MA_0.15_FA_0.8_PbBr_0.75_I_2.25_ | 2024 | 22.31 | 1.250 | 23.05 | ^[16]^ |
| Cs_0.05_(FA_0.77_MA_0.23_)_0.95_Pb(I_0.77_Br_0.23_)_3_ | 2024 | 22.96 | 1.220 | 22.77 | This work |

**References**

[1] K. Lejaeghere, V. Van Speybroeck, G. Van Oost, S. Cottenier, *Critical Reviews in Solid State and Materials Sciences* **2014**, 39, 1.

[2] a)Y. Zhang, J. Sun, J. P. Perdew, X. Wu, *Physical Review B* **2017**, 96, 035143; b)D. G. A. Smith, L. A. Burns, K. Patkowski, C. D. Sherrill, *Journal of Physical Chemistry Letters* **2016**, 7, 2197.

[3] R. Nelson, C. Ertural, J. George, V. L. Deringer, G. Hautier, R. Dronskowski, *Journal of Computational Chemistry* **2020**, 41, 1931.

[4] E. Caldeweyher, S. Ehlert, A. Hansen, H. Neugebauer, S. Spicher, C. Bannwarth, S. Grimme, *Journal of Chemical Physics* **2019**, 150, 154122.

[5] A. Al-Ashouri, E. Kohnen, B. Li, A. Magomedov, H. Hempel, P. Caprioglio, J. A. Marquez, A. B. M. Vilches, E. Kasparavicius, J. A. Smith, N. Phung, D. Menzel, M. Grischek, L. Kegelmann, D. Skroblin, C. Gollwitzer, T. Malinauskas, M. Jost, G. Matic, B. Rech, R. Schlatmann, M. Topic, L. Korte, A. Abate, B. Stannowski, D. Neher, M. Stolterfoht, T. Unold, V. Getautis, S. Albrecht, *Science* **2020**, 370, 1300.

[6] Y. Zheng, X. Wu, J. Liang, Z. Zhang, J. Jiang, J. Wang, Y. Huang, C. Tian, L. Wang, Z. Chen, C.-C. Chen, *Advanced Functional Materials* **2022**, 32, 2200431.

[7] K. Xu, A. Al-Ashouri, Z.-W. Peng, E. Koehnen, H. Hempel, F. Akhundova, J. A. Marquez, P. Tockhorn, O. Shargaieva, F. Ruske, J. Zhang, J. Dagar, B. Stannowski, T. Unold, D. Abou-Ras, E. Unger, L. Korte, S. Albrecht, *Acs Energy Letters* **2022**, 7, 3600.

[8] H. Guo, Y. Fang, Y. Lei, J. Wu, M. Li, X. Li, H. B. Cheng, Y. Lin, P. J. Dyson, *Small* **2023**, 19.

[9] N. Yan, Y. Gao, J. Yang, Z. Fang, J. Feng, X. Wu, T. Chen, S. Liu, *Angewandte Chemie-International Edition* **2023**, 62.

[10] Z. Li, X. Sun, X. Zheng, B. Li, D. Gao, S. Zhang, X. Wu, S. Li, J. Gong, J. M. Luther, Z. a. Li, Z. Zhu, *Science* **2023**, 382, 284.

[11] X. Hu, F. Yao, C. Wang, H. Cui, P. Jia, S. Du, S. Zhou, H. Guan, Q. Lin, W. Ke, C. Tao, G. Fang, *Chemical Engineering Journal* **2024**, 489, 151379.

[12] X. Li, Y. Li, Y. Feng, J. Qi, J. Shen, G. Shi, S. Yang, M. Yuan, T. He, *Advanced Materials* **2024**, 36.

[13] X. Ji, S. Zhang, F. Yu, H. Zhang, L. Zhan, Y. Hu, W.-H. Zhu, Y. Wu, *Science China-Chemistry* **2024**, 67, 2102.

[14] L. Yang, Z. Fang, Y. Jin, H. Feng, B. Deng, L. Zheng, P. Xu, J. Chen, X. Chen, Y. Zhou, C. Shi, W. Gao, J. Yang, X. Xu, C. Tian, L. Xie, Z. Wei, *Adv Mater* **2024**, 36, e2311923.

[15] S. Yang, J. He, Z. Chen, H. Luo, J. Wei, X. Wei, H. Li, J. Chen, W. Zhang, J. Wang, S. Wang, G. Yu, *Advanced Materials* **2024**.

[16] T. Huang, F. Xu, J. Hu, J. Wu, S. Li, P. Chen, X. Jia, Q. Li, H. Yan, Y. Ji, D. Luo, D. Wang, J. Hu, H.-H. Chen, Z. Lu, H. Xu, L. Li, R. Sha, Q. Zhong, X. Bai, M. I. Dar, T. Song, Z. Li, X. Yang, L. Zhao, Z.-H. Lu, Q. Gong, R. Zhu, *Energy & Environmental Science* **2024**, 17, 5984.
